# CdTe Based Energy Resolving, X-ray Photon Counting Detector Performance Assessment: The Effects of Charge Sharing Correction Algorithm Choice

**DOI:** 10.3390/s20216093

**Published:** 2020-10-27

**Authors:** Oliver L. P. Pickford Scienti, Jeffrey C. Bamber, Dimitra G. Darambara

**Affiliations:** Joint Department of Physics, Institute of Cancer Research and Royal Marsden NHS Foundation Trust, London SM2 5NG, UK; jeff.bamber@icr.ac.uk (J.C.B.); dimitra.darambara@icr.ac.uk (D.G.D.)

**Keywords:** energy resolving, photon counting, spectral imaging, CdTe, charge sharing correction, charge sharing effects

## Abstract

Most modern energy resolving, photon counting detectors employ small (sub 1 mm) pixels for high spatial resolution and low per pixel count rate requirements. These small pixels can suffer from a range of charge sharing effects (CSEs) that degrade both spectral analysis and imaging metrics. A range of charge sharing correction algorithms (CSCAs) have been proposed and validated by different groups to reduce CSEs, however their performance is often compared solely to the same system when no such corrections are made. In this paper, a combination of Monte Carlo and finite element methods are used to compare six different CSCAs with the case where no CSCA is employed, with respect to four different metrics: absolute detection efficiency, photopeak detection efficiency, relative coincidence counts, and binned spectral efficiency. The performance of the various CSCAs is explored when running on systems with pixel pitches ranging from 100 µm to 600µm, in 50 µm increments, and fluxes from 10^6^ to 10^8^ photons mm^−2^ s^−1^ are considered. Novel mechanistic explanations for the difference in performance of the various CSCAs are proposed and supported. This work represents a subset of a larger project in which pixel pitch, thickness, flux, and CSCA are all varied systematically.

## 1. Introduction

X-ray photon counting spectral imaging (x-CSI) is currently a hot topic in the medical research field due to the potential of this technique to reduce patient doses in routine procedures [1,2] as well as to provide better material differentiation than dual energy computed tomography (CT) [3]. Further, due to its photon counting nature, x-CSI promises to produce images free from electronic noise [4] that can be used to derive quantitative spectral information rather than just qualitative data with a greater accuracy and less noise than dual energy CT [5]. This opens the possibility that X-ray systems will be able to move beyond the largely structural images they currently deliver and additionally enter the field of molecular imaging [6,7]. The ability of an x-CSI detector to do this will depend on how efficiently it can absorb incident photons and how accurately it can resolve their energy.

In order to maximise the energy resolution of an x-CSI detector, semiconductor materials are often proposed as the conversion material over scintillators, due to the lower noise associated with the direct conversion of photons to electrical current as opposed to via an optical intermediary. Of the various materials proposed, CdTe and CdZnTe (CZT) have drawn particular attention, due to their low pair production energy, high charge carrier mobility and high absorption efficiency across the range of energies commonly used for medical X-ray imaging (at 100 keV, 1 mm of CdTe has an absorption efficiency of ~80%, compared with ~35% for Ge and ~4% for Si at the same thicknesses) [8]. Historically CZT has suffered from polarization at the X-ray fluxes used in medical CTs [9]; however, in recent years this problem has been addressed with some success, resulting in materials with good enough stability for high flux medical applications [10]. The limiting factor in achieving photon counting clinically with these semiconductor materials has thus shifted to the rate at which the electronics on which the detectors are based can count and reset, with this constraint setting an upper limit on the pixel sizes that can be achieved [11].

In an attempt to improve the spatial resolution and reduce the required count rates on a per pixel basis, some groups have opted to move to smaller pixel pitches [12], resulting in a lower flux per pixel. This has the added benefit of improving spatial resolution of the system and is made possible by the photon counting system’s ability to effectively eliminate false counts that would otherwise be due to electronic noise [4]. There are drawbacks to this approach however, including the increasing prevalence of charge sharing effects (CSEs) as pixel sizes are reduced, degrading the spectral performance of the detector. CSEs refer to any mechanism which result in the energy from a single incident photon being deposited as charge across multiple pixels and includes inter-pixel charge cloud diffusion, Compton scattering and X-ray fluorescence. In order to reduce the effect of CSEs whilst still maintaining small pixels sizes, research groups have proposed a variety of charge sharing correction algorithms (CSCAs).

A wide range of CSCAs have been proposed [13,14,15,16]; however, this work will focus on those implemented on chip. These CSCAs can broadly be divided into online techniques and offline techniques. Online techniques are more common and attempt to identify the various pixels a given photon has interacted with and then to reconstruct its energy from the signals, either pre or post-thresholding, and assigning it to a single pixel [17,18,19] during data acquisition. Offline CSCAs in contrast record how many CSEs are suspected at each pixel intersection and then attempt to correct for the likely spectral distortions of this offline [20]. Whilst in developing these CSCAs the performance of a test system both with and without CSCA is often published, few publications have looked to compare the various CSCAs directly. Similarly, whilst literature exists investigating the effects of physical detector parameters such as pixel thickness or pitch on photon counting performance [21,22,23], these often consider only the case of a single X-ray flux, a single thickness, or the case where no CSCA is present. Further, comparisons between the works are often difficult due to the different simulation schemes, assumptions, fluxes, and metrics used between them. Due to the wide range of X-ray fluxes over which medical X-ray imaging takes place; the various task specific demands on performance; and the growing number of CSCAs proposed, there is a need for a deeper understanding of the interplay between these various factors. Our current work attempts to fill this gap in knowledge by systematically evaluating x-CSI detector performance, with respect to a range of metrics, such as pixel pitch, thickness, flux, and CSCA choice. In doing so it reveals and explains general trends in behaviour of CSCAs with pixel pitch and flux and allows for different classes of CSCA to be compared.

In total 715 different x-CSI detector designs are compared at four different medically relevant fluxes in order to detect and explain general trends in detector behaviour and to allow for more informed decision making in x-CSI detector prototype design. This set includes all combinations of 11 pitches (100 µm–600 µm, in 50 µm increments), 5 thicknesses (1 mm–3 mm, in 0.5 mm increments) and 12 different CSCAs, for a total of 2860 separate simulations. Due to the large number of variables in this investigation, analysis of all parameters simultaneously would be unwieldly. For this reason, the results are divided into smaller publications in which one or more parameters are held constant. The work presented in this paper forms part of this larger study and considers the behaviour of six pre-thresholding CSCAs with the pixels of the same size without a CSCA. Results are presented as a function of pitch for all pitches considered, with respect to four different metrics. This paper primarily concerns itself with CSCA behaviour at 10^7^ photons mm^−2^ s^−1^; however, results from 10^6^ and 10^8^ photons mm^−2^ s^−1^ are presented where they help to clarify the mechanisms behind the observed trends.

It is hoped that this paper will be of interest to the wider spectral X-ray photon counting community, and of particular benefit to those seeking to design pre-thresholding CSCAs.

## 2. Materials and Methods

Unless otherwise stated, all simulations presented in this work were performed using our inhouse x-CSI simulation framework called CoGI (Comsol-Gate Interlocutor), which has been previously presented, detailed, and validated [24,25]. CoGI works by using a combination of Monte Carlo, finite element method, and custom codes to simulate the imaging chain of an x-CSI system up to the signal readout stage. The Monte Carlo component is based on the open source GATE software [26] and is responsible for generating the incident photons and calculating the photon–matter interactions that determine when and where charge is deposited within the CdTe sensor material. The finite element method components of the framework are performed using the commercial software COMSOL [27] and are responsible for calculating the charge induction efficiency (CIE) values for charges deposited in the sensor material as a function of time and the charge’s location within the pixel volume. The custom codes are executed in Matlab and are referred to collectively as “Supervisory Gate Scripts” (SGS). These codes cross reference the outputs from the Monte Carlo and finite element components and apply any CSCAs selected by the user to produce a list containing the output signals in terms of pixel number, time, and intensity. The current incarnation of SGS is capable of modelling a range of different CSCAs, as will be detailed below. Further details may be found in an earlier paper [25], which also provides a validation of this framework.

### 2.1. Monte Carlo Parameters

The simulations performed in this work were all run with the same random seed so that the performance of the different CSCAs and geometries could be compared on exactly the same incident photons. The primary parameters needed by CoGI to define the Monte Carlo component of the simulations are the physical geometry of the setup and the irradiation parameters.

The physical geometry modelled here involved a single crystal of CdTe with a cross-section of 21 mm × 21 mm and a variable thickness, T. T was systematically increased from 1 mm to 3 mm in 0.5 mm steps across the various simulations. This range was chosen as it encompasses the range of CdTe thicknesses currently under consideration in the wider community looking at photon counting CT with CdTe [28]. The X-ray source was defined as a 24 mm × 24 mm square, parallel to the sensor material and with their centres in alignment.

The photons that comprise the irradiation were defined to be monoenergetic at 80 keV and to be normally incident to the sensor material. Their point of origination within the X-ray source was randomly determined with a rectangular distribution covering the whole source. Considerations in determining the energy of the photons included that it should be high enough to ensure good spectral separation between the main photopeak, the escape peak, and the Cd fluorescence X-ray peak, whilst still remaining at an energy relevant to medical CT examinations. We selected 80 keV because it meets these requirements and is also approximately the k-edge for gold, which is of interest to our group’s research activities.

Each physical geometry defined was irradiated sequentially with an X-ray source flux of ~ 10^6^, 10^7^, 10^8^, and 10^9^ photons mm^−2^ s^−1^. The duration of each irradiation was varied so as to keep the total number of incident photons constant between the various fluxes. The simulated irradiation time was bounded both by the real-world time the simulations would take (upper limit) and the need to keep uncertainty due to the Poisson statistics of the simulation reasonable (the lower limit). In practice, this meant a simulated time of 10 ms for a flux of 10^6^ photons mm^−2^ s^−1^, 1 ms for a flux of 10^7^ photons mm^−2^ s^−1^ and 100 µs for a flux of 10^8^ photons mm^−2^ s^−1^. In all cases the simulated time was still orders of magnitude greater than the shaping time of the systems simulated (which were of the order of 10 s of ns). The result was that the minimum number of photons involved in photoelectric energy transfers across the various sensors modelled was ~1.7 million.

### 2.2. Finite Element Parameters

The adjoint continuity equations of Prettyman [29] were used to calculate the CIE values of a range of regularly space points within CdTe pixels of the various thickness and pitch combinations considered in this work, as described in previous publications [24,30,31]. Note that due to the large number of geometries simulated in this work the CIE maps for centre pixels will be applied to all pixels, neglecting the electric field deviations for corner and edge pixels considered in the previous publications [25]. CIE in this context is defined as

CIE = q/Q (1)

where Q is the free charge in the sensor volume and q is the charge induced at the collecting electrode due to the movement of these free charges. CoGI calculates CIE maps for electrons and holes separately and then forms a composite map by summing the contributions from each on a point-by-point basis. The result is a map of CIE as a function of intrapixel coordinates that includes the contribution of both electrons and holes. The material properties for the CdTe used in this simulation can be seen in Table 1. The bias voltage, pixel spacing and shaping time for the simulations were selected based on our experience with a prototype photon counting detector we have experience with in our lab (an Actaeon series pixilated photon counting detector, manufactured by XCounter, which also utilized CdTe as a sensor material). The exact details of this system are covered by a non-disclosure agreement so will not be discussed further here. Pixel pitches of 100 µm–600 µm were modelled, in 50 µm steps, again consistent with the majority of the systems currently used in the field [28]. The pad spacing was held constant across the various geometries, however the anode size and bias voltage were varied to keep the shaping time constant. The voltage and CdTe thickness used in the prototype system, V_α_ and T_α_, respectively, were used to calculate the voltage needed for the various sensor thicknesses according to the equation

V_X_ = V_α_ (T_X_/T_α_)^2^ (2)

where V_X_ is the voltage needed for a sensor of thickness T_X_.

### 2.3. Supervisory Gate Script Parameters

The Monte Carlo simulation output was fed into SGS to be pixelated at the appropriate pitch by converting global spatial coordinates into intrapixel coordinates. Handling pixelization in this way has the dual benefits of reducing the computational overhead of the Monte Carlo simulation and also more representing the CdTe crystal as a continuous block rather than as a pixelated entity. The latter point removes the need to assess photon absorption probability at interpixel boundaries within the continuous CdTe volume, which could otherwise lead to sources of error if not implemented correctly. Each energy depositing interaction in the list was then assigned a CIE value based on a trilinear interpolation of the relevant CIE maps from the finite element method, resulting in a list of pre-thresholded signals. SGS then used the rules of a given CSCA (or the no CSCA rules) to run through the list and identify groups of events that need to be summed into a single readout event. The total signal produced by these events is then compared to the preset energy thresholds so that the event can be assigned to the correct energy bin. In a physical system all counters that the event passes would be incremented by one and then the number of events in each bin determined by subtraction, however due to the large amount of data being processed in this work the more computationally expedient approach of assigning signals directly to their correct energy bin was adopted. The resulting data set contains for each pixel a list of output signal intensities and times, which can be used to construct an image or aggregated to assess the performance of the detector as a whole. Due to the flat field irradiation used, the aggregated readout scheme was used in this work.

### 2.4. Charge Sharing Correction Algorithms Considered

All CSCAs considered in this work function in a similar way, by identifying events that occur in nearby pixels within some time window and summing them together to form a single event. The theory behind this approach is that events that are spatially and temporally close together are more likely to result from a single photon generating charge across multiple pixels than to originate from multiple independent photons. By summing these events together then it is hoped that the original energy of the incident photon can be reconstructed. The CSCAs considered in this work were classified with respect to two terms: neighbourhood size and neighbourhood locality.

Neighbourhood size (NS) refers to the size of the search area, in pixels, over which events were identified for summing. 2 × 2 CSCAs consisted of a 2-pixel by 2-pixel search area, whilst 3 × 3 CSCAs utilized a 3-pixel by 3-pixel search area.

**Figure 1:**
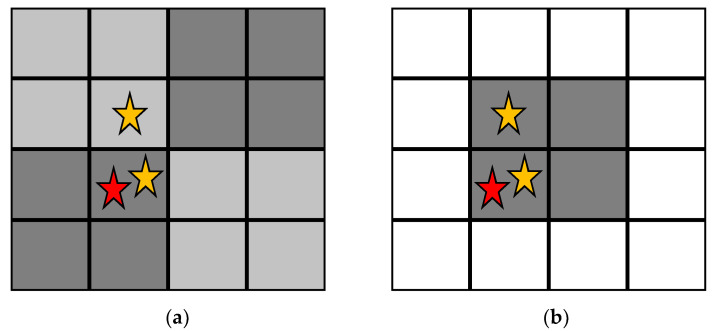
A 16 pixel array containing 3 detected events, with the red star representing the first of these events to be detected. (**a**) Static charge sharing correction algorithms (CSCAs) predefine the various search areas (shades of grey) before any events are detected. (**b**) In contrast, dynamic CSCAs define search areas after an event is detected based on a predetermined rule. In this case, the dynamic 2 × 2 CSCA is being applied, so the 2 × 2 search area is defined such that the first detected event is in the bottom left pixel of the search area.

Neighbourhood locality (NL) refers to the way in which the search area (neighbourhood) is defined, as shown in Figure 1. Static CSCAs use a predefined scheme to link all pixels to a unique NxN neighbourhood of pixels that does not change throughout the irradiation. In contrast, dynamic CSCAs define the NxN neighbourhood relevant to each event only when that event is detected, using a predefined rule. For the dynamic 2 × 2 CSCA in this work that rule is that the event is placed in the bottom left pixel of a 2 × 2 search area. For the dynamic 3 × 3 CSCA in this work the rule is that the event is placed in the middle pixel of a 3 × 3 search area. Due to this on-the-fly neighbourhood construction, dynamic CSCAs may lead to a case where proposed neighbourhoods would overlap. Pixels called multiple times in quick succession like this report their full charge to the first neighbourhood they are assigned to, but a charge of zero to all subsequent calls, until they have reset and are ready to detect another event.

**Figure 2:**
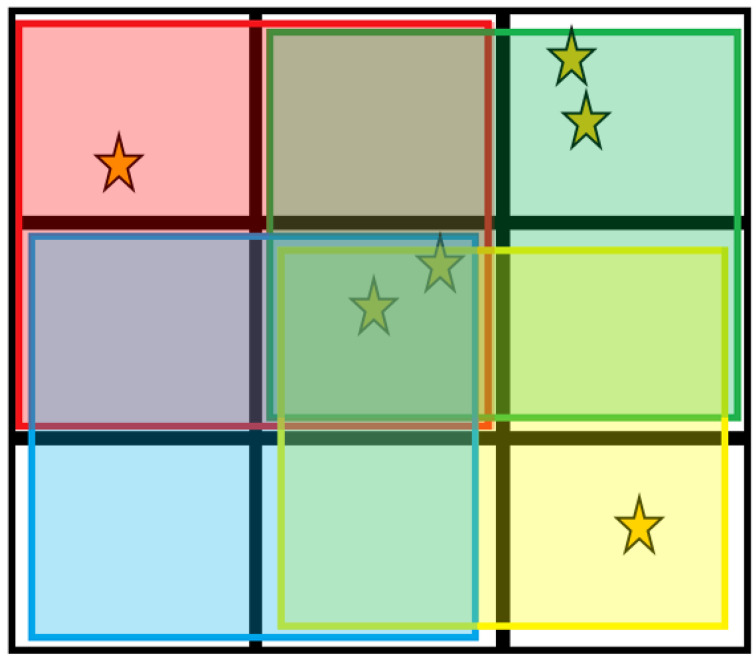
The Hybrid CSCA works by first constructing a 3 × 3 search area according to the rules of the Neighbourhood locality (NL) type to which it belongs. Next it constructs 4 overlapping 2 × 2 sub-search areas within the initial 3 × 3 search area. The total charge in each sub-search area is compared, with the highest total being assigned to the output. In this example, assuming all stars represent the same amount of charge, the green search area (top right) would determine the output as it contains the highest total charge (4, compared with 3 for yellow (bottom right) and red (top left), and 2 for the blue (bottom left)).

A final class of CSCA considered in this work is referred to here as a Hybrid CSCA. CSCAs can again be dynamic or static in nature, however their NS is both 3 × 3 and 2 × 2 at different stages in the reconstruction. When an event is detected and a Hybrid CSCAs is being implemented, first a 3 × 3 search area is defined which contains the event (depending on the NL mode in operation). Once this is done, four 2 × 2 sub-search areas are defined which cover this region (see Figure 2). The events in each sub-search area are then summed and the results for the four sub-search areas compared. The sub-search area which has the largest signal is used to generate the output, with any events within the 3 × 3 search area but not the winning 2 × 2 sub-search area being discarded. Table 2 lists all of the CSCAs used in this work, along with their shorthand labels used in the figures.

### 2.5. Setting Energy Bin Thresholds

In order to better model the results expected from a physical x-CSI detector, the continuous energy spectrum of events needed to be binned according to some preset thresholds. The major spectral features expected for a monoenergetic irradiation of CdTe are the primary photopeak, the escape peak and an X-ray fluorescence peak. To ensure these features could be isolated into its own bin, and in keeping with a feasible number of bins based on the current state of the field, 4 energy thresholds were chosen for this work. The thresholds for each bin were determined by exhaustive investigation of “high resolution” energy spectra (100 energy bins) from the various geometries studied, and cross-referenced Monte Carlo data. This investigation revealed that photons of energy E keV that were fully absorbed in the detector would often register signals < E keV but would almost never register as an energy above (E + 1.5) keV. With this in mind, the following bin thresholds were defined:

1st threshold: 10 keV. This was set as the noise flood of the system, meaning the energy above which a post reconstruction event needs to rise to trigger a count in the detector. Events above this threshold but below the 2nd threshold will be assigned to this bin, which should contain the Cd X-ray fluorescence peaks (at ~23 and ~26 keV).

2nd threshold: 30 keV. Events above this threshold and below the 3rd threshold will be assigned to this bin, which should contain most of the events from the escape peak.

3rd threshold: 60 keV. Events above this threshold but below the 4th threshold will be assigned to this bin. This bin should contain the full-energy photopeak.

4th threshold: 83 keV. All events above this threshold will be assigned to this bin. As this threshold is set above the high energy tail observed for the full-energy photopeak it should only contain events which have resulted from more than one incident photon being summed together. This bin will thus act as a proxy for measuring the amount of pulse pileup occurring in the system.

### 2.6. Metrics Used to Assess Detector Performance

As noted above, the binned counts from all of the pixels in a given simulation were aggregated together for analysis. These bins were then used to define the following metrics by which the various detector-CSCA combinations would be compared:

Absolute Detection Efficiency (ADE): The ratio of detector counts to the number of incident photons.

(3) $$\mathrm{ADE}=\overset{4}{\underset{E=1}{\sum }}B_{E}/I$$

where *I* is the number of incident photons and *B_E_* is the number of counts in energy bin *E*.

Absolute Photopeak bin Efficiency (APE): The fraction of incident photons that are detected in the photopeak bin.

(4) $$\mathrm{APE}=B_{3}/I$$

where *B*_3_ is the number of counts in the photopeak bin.

Relative Coincidence Counts (RCC): The fraction of recorded counts that are above the 4th threshold

(5) $$\mathrm{RCC}=B_{4}/\overset{4}{\underset{E=1}{\sum }}B_{E}$$

where *B*_4_ is the number of counts in the coincidence bin.

Binned Spectral Efficiency (BSE): The fraction of recorded counts that are in the photopeak bin.

(6) $$\mathrm{BSE}=B_{3}/\overset{4}{\underset{E=1}{\sum }}B_{E}$$

### 2.7. Theoretical Absolute Detection Efficiency Model

APE, RCC, and BSE all have easily defined maximal performance values (100% for APE and BSE, 0% for RCC); however, this is not the case for ADE. ADE is a metric designed primarily for x/γ-ray spectroscopy with single collecting anodes rather than pixelated anodes, though it can be used with the later if care is taken. To understand the difference this makes, consider a single photon that deposits its energy at multiple locations within the crystal due to Compton scattering. A single anode would sum the charge of these events into a single count, whilst a pixelated array could register multiple counts if the interactions occurred above different anode pixels. If each photon shared its energy across two pixels for example, then the maximum ADE value would be 200%: 100% of the incident photons being absorbed and each producing 2 counts. CSCAs are designed to correct for one photon triggering multiple pixels, and so would be expected to bring the maximum ADE value back to 100%. CSCAs are not perfect however, and may mistakenly sum two unrelated counts, leading to a lower maximum ADE value. Higher ADEs can thus result from detecting more incident photons correctly or from failing to correct for CSEs, meaning that a single incident photon causes multiple counts in the detector. Similarly, a low ADE value may indicate better CSE correction, or it may indicate more counts lost due to incorrect summing. For ADE therefore we need an idealised value to compare against, in which CSEs are either perfectly corrected for or else never exist in the first place. This value will be referred to as the “Theoretical ADE” in this work. It is calculated analytically using the following approach:

1. Calculate the probability of an incident photon photoelectrically interacting with the detector such that it produces a count in the detector, P_I_, using the equation
  P_I_ = 1 − e^−MρT^ (7)
  where ρ is the density of CdTe, T is the pixel thickness, and M is the mass attenuation coefficient.
2. Calculate the number of incident photons per pixel per second, F_pix_, using the equation
  F_pix_ = F_det_ A (8)
  where F_det_ is the flux incident on the detector (in units of photons mm^−2^ s^−1^) and A is the area of pixel with the pitch being investigated.
3. The rate at which incident photons interact with the sensor material, R_I_, is then given by the equation
  R_I_ = F_pix_ P_I_ (9)
4. For an idealised detector with no charge sharing we assume photon interactions deposit their energy at a single point in space and time. Assuming that the photon interactions with a given pixel follow a Poisson distribution, we can calculate the probability that two unrelated photons interact with the same pixel by chance within a time window t_w_. This probability, P_T_, is given by the equation
  P_T_ = 1 − exp(−R_I_t_W_) (10)
5. When t_w_ represents the time required for two photons to be separately resolved by the detector, the number of uniquely detectable photon interactions, U, is given by
  U = R_I_ (1 − P_T_) (11)
  This approximation models a paralysable detector setup; however, the detectors in this work are non-paralysable, due to the reset triggers simulated. Equation (11) is thus not valid for high values of P_T_, however as the P_T_ values considered in this work vary between 0.003 and 0.1, this equation should give an approximation sufficiently accurate for comparing the performance of the different CSCAs. It should again be stated at this point that the point of the current work is a qualitative comparison of the behaviours of the various CSCAs, and for this goal the estimate from Equation (11) is reasonable.
6. Theoretical ADE values are then determined for this idealized detector according to the equation
  ADE = U/F_pix_ (12)
7. Steps 1–6 are then repeated for each pixel pitch and flux considered in this work, to give a theoretical ADE value for each detector that can be used as a point of reference. CSCAs which produce ADE values above this theoretical ADE are imperfectly correcting for CSEs whilst those that produce ADE values below the theoretical value are amplifying the effects of pulse pileup.

### 2.8. Division of Results for Analysis

As mentioned in the introduction, the work detailed in this paper represents a subset of a larger study in which the detector performance was assessed over four degrees of freedom (thickness, pitch, flux, and CSCA). The large data set, and multiple degrees of freedom, make analysis of the entire dataset in a single paper unwieldly and for that reason the analysis is divided across several publications. The work in this paper represents the second such submission, with the first focusing on pixel pitch and thickness at a single flux, without using a CSCA [32]. That work explained the behaviour of the “No CSCA” data for each metric in terms of CSEs and pulse pileup. Relevant conclusions from that work will be summarized and used to explain the performance of the various CSCAs considered in this paper. These CSCAs will be evaluated at all simulated pitches, but only at a single pixel thickness (1.5 mm) and primarily at a single flux (10^7^ photons mm^−2^ s^−1^) in order to keep the size of the analysis manageable. Results from higher or lower fluxes may be shown where doing so helps to support or clarify the proposed mechanisms responsible for observed CSCA behaviours.

## 3. Results

The discussion of the generated data will focus on a single flux (10^7^ photons mm^−2^ s^−1^) to make establishing and explaining trends simpler, however demonstrating the mechanisms responsible for the observed trends can often be assisted by showing data from a higher or lower fluxes, to emphasise the roles of pulse pileup or CSEs, respectively. For this reason, results for some metrics are presented at multiple flux levels. To avoid confusion, the minimum number of additional fluxes are used, meaning that for some metrics two or three fluxes are presented whilst for other metrics only one is needed.

### 3.1. Absolute Detection Efficiency

**Figure 3:**
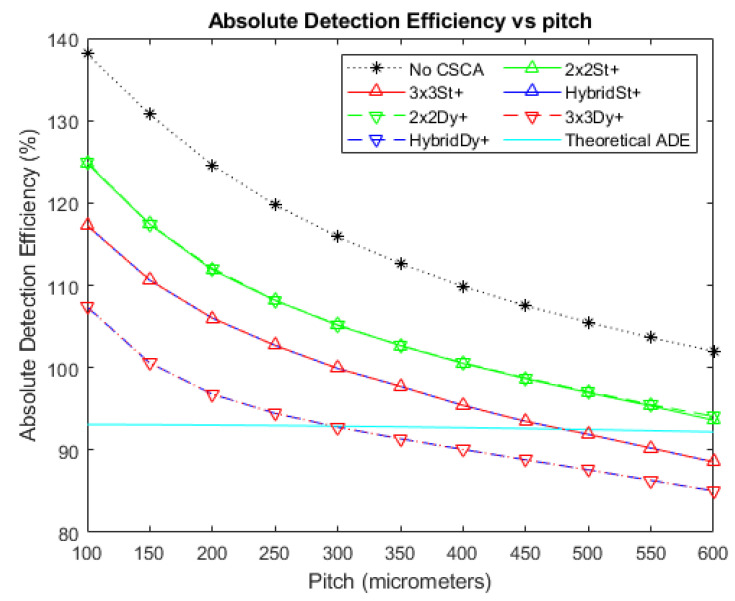
A plot of Absolute Detection Efficiency (ADE) versus pixel pitch at an incident photon flux of 10^6^ photons mm^−2^ s^−1^. The Hybrid and 3 × 3 CSCAs perform indistinguishably according to this metric, resulting in significant overlap. Similarly, the 2 × 2 dynamic and static CSCAs overlap almost entirely at this flux level. The case where no CSCA is applied is also shown, along with the theoretical ADE value proposed earlier as a reference.

**Figure 4:**
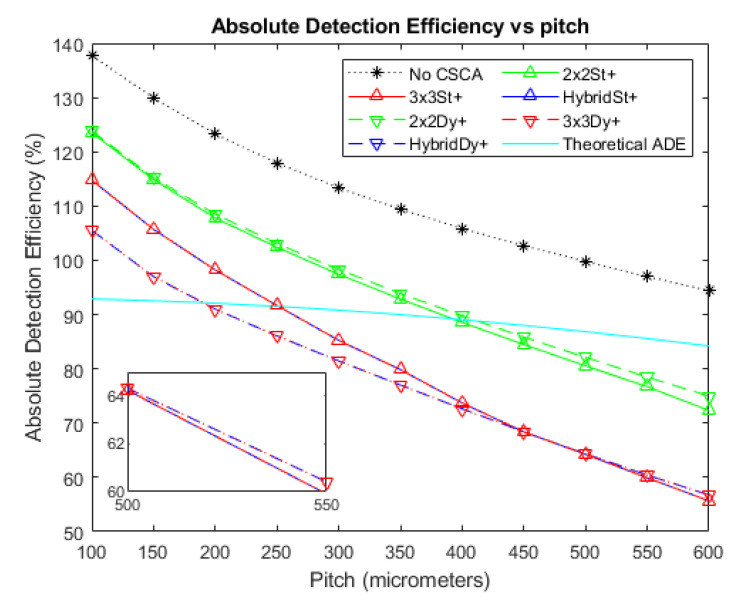
A plot of ADE versus pixel pitch at an incident photon flux of 10^7^ photons mm^−2^ s^−1^. The Hybrid and 3 × 3 CSCAs perform indistinguishably according to this metric, resulting in significant overlap. Figure inset shows that the dynamic and static CSCAs swap order at the highest pitches considered in this figure. The case where no CSCA is applied is also shown, along with the theoretical ADE value proposed earlier as a reference.

**Figure 5:**
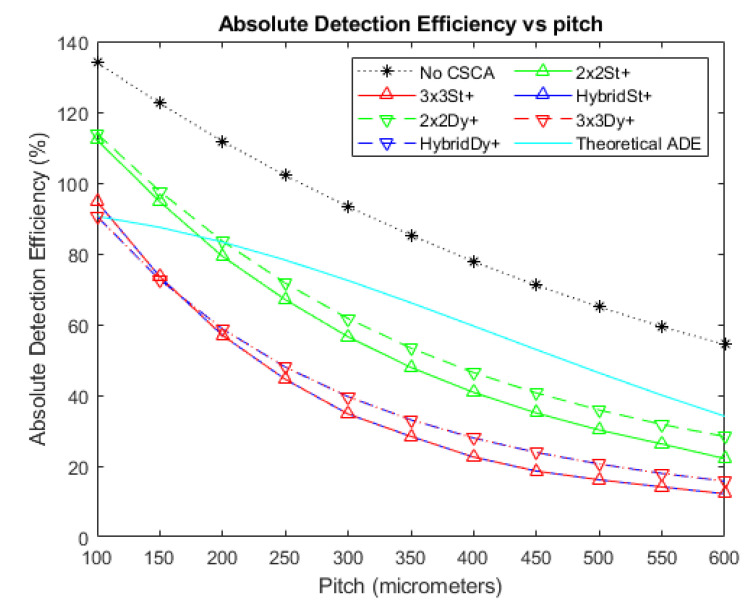
A plot of ADE versus pixel pitch at an incident photon flux of 10^8^ photons mm^−2^ s^−1^. The Hybrid and 3 × 3 CSCAs perform indistinguishably according to this metric, resulting in significant overlap. The case where no CSCA is applied is also shown, along with the theoretical ADE value proposed earlier as a reference.

Figure 3, Figure 4 and Figure 5 show the ADE produced by the various CSCAs as a function of pixel pitch at photon fluxes of 10^6^, 10^7^, and 10^8^ photons mm^−2^ s^−1^, respectively. Further shown on this graph are the ADE values produced when no CSCA is applied (the No CSCA case) and the theoretical ADE value calculated analytically when pulse pileup is included but CSEs are neglected. The first point to note is that the No CSCA case results in higher ADE values than the theoretical ADE, due to the presence of CSEs which can lead to multiple counts from a single incident photon. All CSCAs lead to a reduction in ADE from the No CSCA case, implying at least some correction of CSEs. It can be seen that 2 × 2 CSCAs behave consistently regardless of the NL method used at low fluxes, though dynamic NL methods outperform static ones at higher fluxes. Moreover, 2 × 2 CSCAs also form a distinct band on the plots, distinct from the 3 × 3 and Hybrid CSCAs. In stark contrast, the 3 × 3 and Hybrid CSCAs perform almost identically to each other with regards to the metric of ADE. For both 3 × 3 and Hybrid CSCAs, the dynamic NL method can be seen to produce lower ADE values at lower per pixel fluxes but higher ADE values at higher per pixel fluxes. Which is closer to the theoretical case depends on the pitch in question.

**Figure 6:**
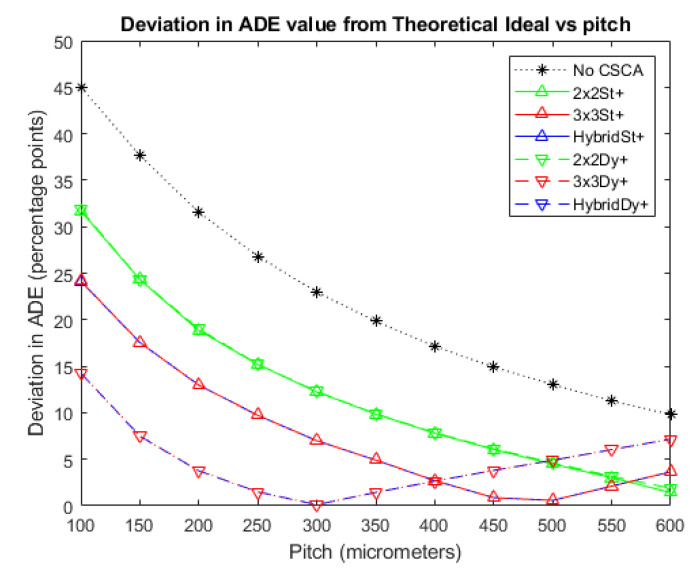
A plot of the difference between each CSCA and the theoretical ADE proposed earlier at an incident photon flux of 10^6^ photons mm^−2^ s^−1^. The difference is taken directly from the data for Figure 3 and so is expressed as percentage points. Again, the case where no CSCA is applied is presented for comparison.

**Figure 7:**
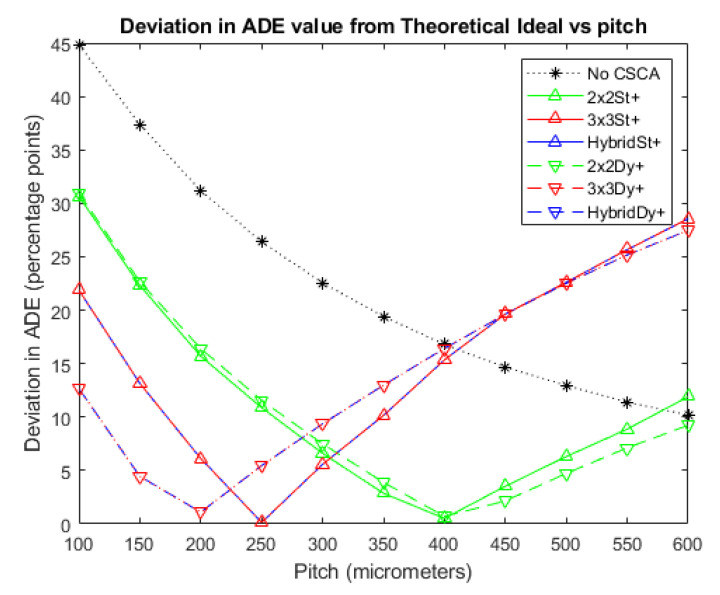
A plot of the difference between each CSCA and the theoretical ADE proposed earlier at an incident photon flux of 10^7^ photons mm^−2^ s^−1^. The difference is taken directly from the data for Figure 4 and so is expressed as percentage points. Again, the case where no CSCA is applied is presented for comparison.

Figure 6 and Figure 7 show the deviations of the various CSCAs from the theoretical expectations as a function of pixel pitch. Deviations are measured using the ADE plots above and so are in units of percentage points rather than percentages. In these plots the lower the deviation noted, the better the fit between CSCA and the expected result for pixels of that size if no CSEs were present. All CSCAs can be seen to have a concave shape, dropping in value with increasing pitch up to a point before beginning to rise again. Both 3 × 3 and Hybrid CSCAs can be seen to reach peak performance in this regard at lower pixel pitches than their 2 × 2 counterparts and also outperform them at low per pixel fluxes. At higher per pixel fluxes however these same CSCAs underperform not just the 2 × 2 but also the No CSCA case.

### 3.2. Absolute Photopeak Efficiency

**Figure 8:**
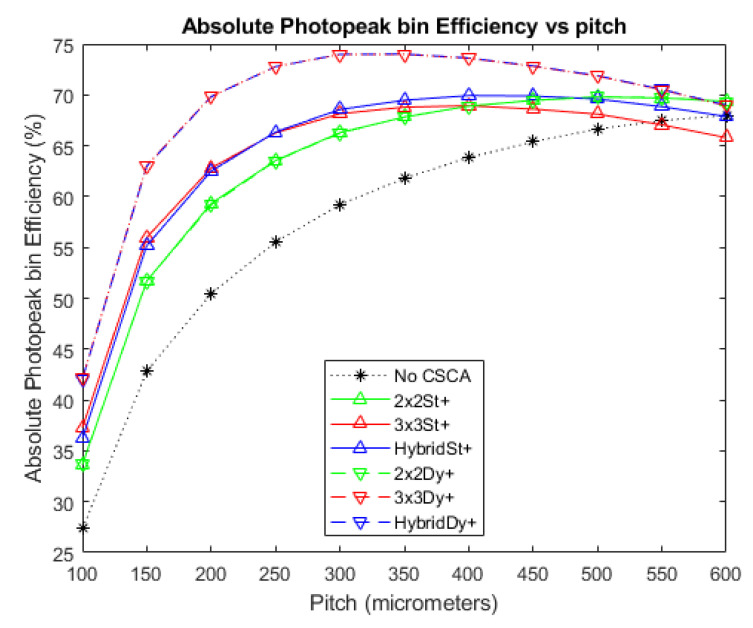
A plot of APE versus pixel pitch at an incident photon flux of 10^6^ photons mm^−2^ s^−1^. The dynamic Hybrid and dynamic 3 × 3 CSCAs perform indistinguishably according to this metric, resulting in significant overlap. Similarly, the 2 × 2 dynamic and static CSCAs overlap almost entirely at this flux level. The case where no CSCA is applied is also shown as a reference.

**Figure 9:**
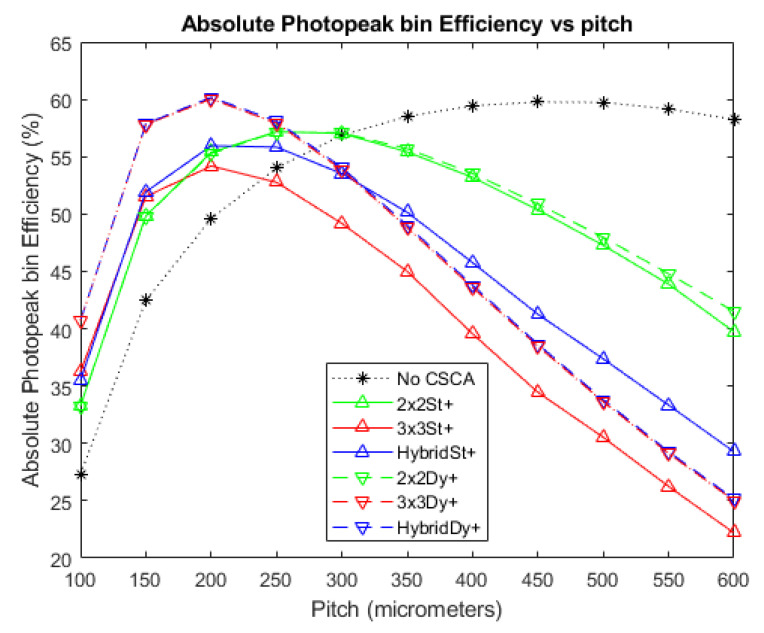
A plot of APE versus pixel pitch at an incident photon flux of 10^7^ photons mm^−2^ s^−1^. The dynamic Hybrid and dynamic 3 × 3 CSCAs perform indistinguishably according to this metric, resulting in significant overlap. Similarly, the 2 × 2 dynamic and static CSCAs overlap at low pitches (below ~350 µm) but begin to become distinguishable at the higher pitches considered here. The case where no CSCA is applied is also shown as a reference.

Figure 8 and Figure 9 show the APE produced when no CSCA is applied, as well as for when the various CSCAs are employed, at photon fluxes of 10^6^ and 10^7^ photons mm^−2^ s^−1^, respectively. The results are again shown as a function of pixel pitch and in this case higher values represent better results. The data show a convex trend, with each CSCA case rising to a peak value before falling again, with this decline resulting in CSCAs underperforming compared with the No CSCA case at higher per-pixel fluxes. All CSCAs reach a peak APE value before the No CSCA case, with the 3 × 3 and Hybrid CSCAs peaking at the lowest pixel pitches. As with Figure 3, Figure 4 and Figure 5, the 2 × 2 dynamic and 2 × 2 static CSCAs are almost identical at lower fluxes, with the dynamic 2 × 2 CSCA outperforming its static counterpart more as per pixel fluxes increase. As was the case with Figure 3, Figure 4 and Figure 5, dynamic 3 × 3 and dynamic Hybrid CSCAs perform indistinguishably; however, in Figure 8 and Figure 9, the static versions of these CSCAs behave differently to one another, with the dynamic Hybrid CSCA dropping in performance more gradually with increasing per-pixel flux than its 3 × 3 counterpart.

### 3.3. Relative Coincidence Counts

**Figure 10:**
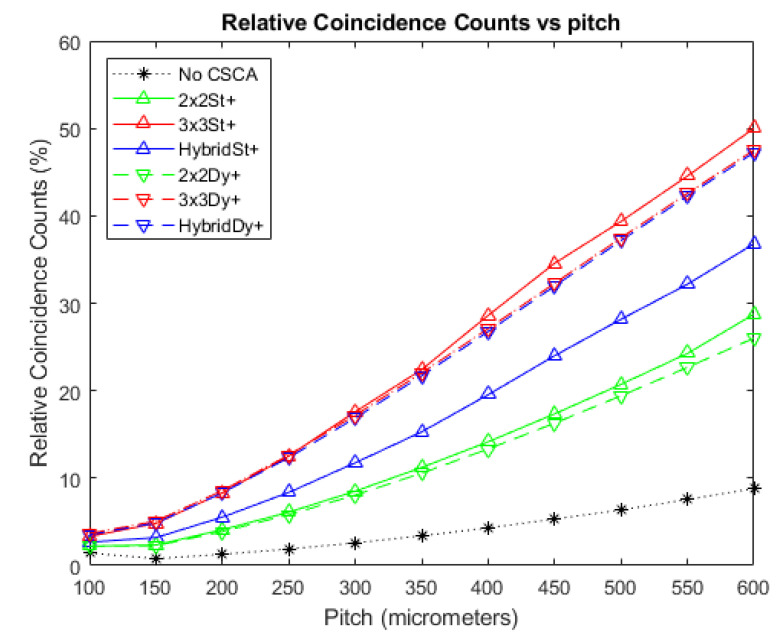
A plot of RCC versus pixel pitch at an incident photon flux of 10^7^ photons mm^−2^ s^−1^. The dynamic Hybrid and dynamic 3 × 3 CSCAs overlap significantly with each other at all pitches, and with the static 3 × 3 CSCA at pitches below ~300 µm. Similarly, the 2 × 2 dynamic and static CSCAs overlap at low pitches (below ~300 µm) but begin to become distinguishable at the higher pitches considered here. The case where no CSCA is applied is also shown as a reference.

Figure 10 shows how the RCC vary as a function of pixel pitch at a photon flux of 10^7^ photons mm^−2^ s^−1^. The case where no CSCA is applied is considered, in addition to data for when the various CSCAs are applied. As coincidence counts can only be produced by pulse pileup, RCC values are a proxy for pulse pileup and consequently a lower value represents less pileup and thus better performance. All CSCAs experience higher RCC values than the No CSCA case, with 2 × 2 CSCAs having lower RCC values than their 3 × 3 and Hybrid counterparts. For both the 3 × 3 and 2 × 2 CSCCAs the dynamic and static NL methods produce similar results at small pixel pitches; however, they increasingly diverge as pixel pitch increases, with dynamic CSCAs outperforming their static counterparts. In contrast, the static Hybrid CSCA performs better than the dynamic Hybrid CSCA at all pitches investigated here, with its advantage growing as pixel pitch increases.

### 3.4. Binned Spectral Efficiency

**Figure 11:**
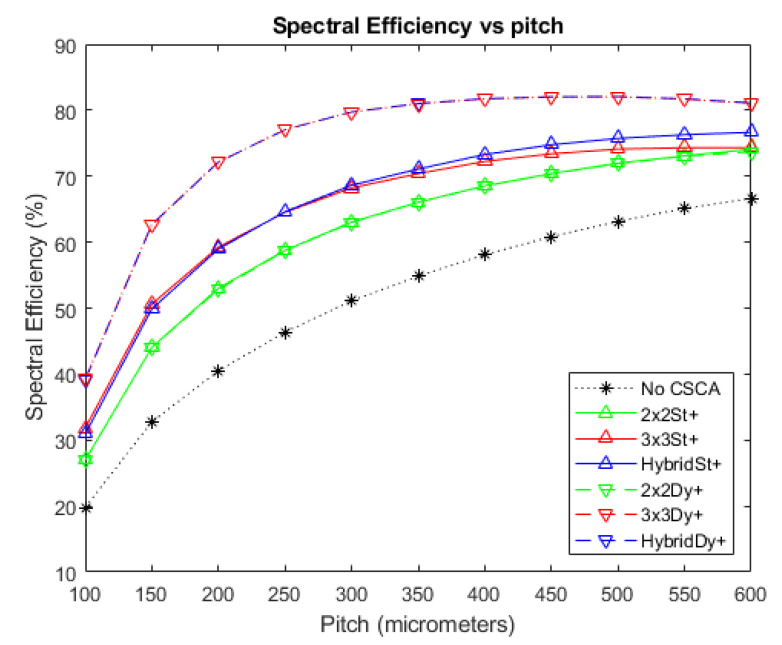
A plot of BSE versus pixel pitch at an incident photon flux of 10^6^ photons mm^−2^ s^−1^. The dynamic Hybrid and dynamic 3 × 3 CSCAs perform indistinguishably according to this metric, resulting in significant overlap. The same is true for the dynamic 2 × 2 and static 2 × 2 CSCAs. Similarly, the static Hybrid and static 3 × 3 CSCAs overlap significantly at lower fluxes but diverge from about ~300 µm. The case where no CSCA is applied is also shown as a reference.

**Figure 12:**
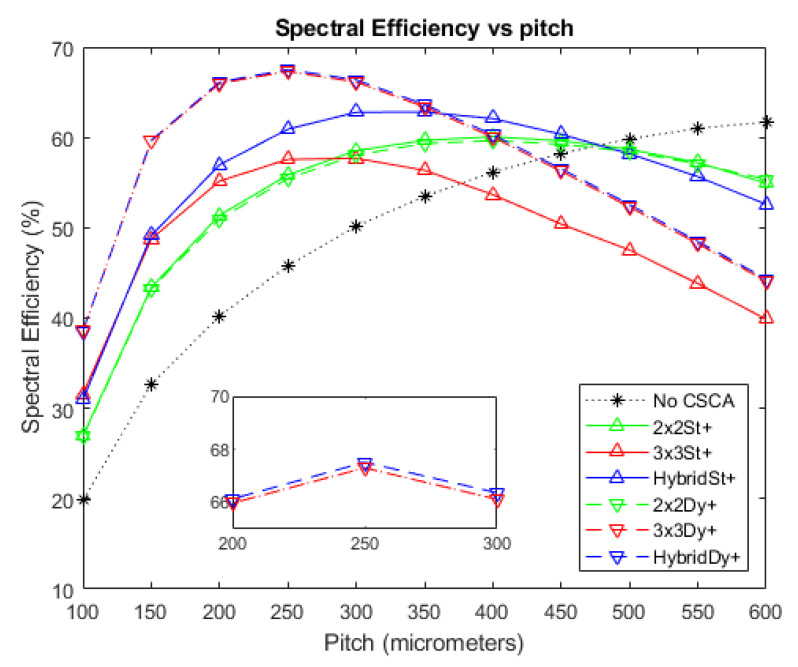
A plot of BSE versus pixel pitch at an incident photon flux of 10^7^ photons mm^−2^ s^−1^. The dynamic Hybrid and dynamic 3 × 3 CSCAs perform exceptionally closely according to this metric (see inset), resulting in significant overlap. The same is true for the dynamic 2 × 2 and static 2 × 2 CSCAs. The case where no CSCA is applied is also shown as a reference.

**Figure 13:**
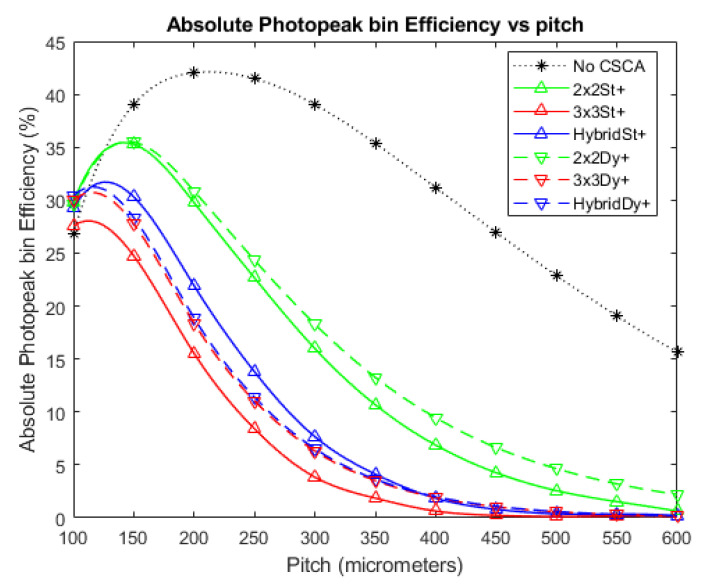
A plot of BSE versus pixel pitch at an incident photon flux of 10^8^ photons mm^−2^ s^−1^. The dynamic Hybrid and dynamic 3 × 3 CSCAs perform exceptionally closely according to this metric, resulting in significant overlap. The case where no CSCA is applied is also shown as a reference.

Figure 11, Figure 12 and Figure 13 show the BSE, as a function of pixel pitch, for the fluxes 10^6^, 10^7^, and 10^8^ photons mm^−2^ s^−1^, respectively. The No CSCA case is shown in addition to values for all of the CSCAs considered in this work. Again, a convex trend is found for the relation between pitch and the metric, though this is most evident at higher fluxes (Figure 12). All CSCAs peak at a lower pixel pitch than the No CSCA case (which looks like it will peak at a pitch just larger than those investigated here), with 3 × 3 and Hybrid CSCAs peaking before their 2 × 2 counterparts. NL selection makes little to no difference for the 2 × 2 CSCAs but has a more pronounced effect for the 3 × 3 and Hybrid cases. Static 3 × 3 and static Hybrid CSCAs both perform similarly at low fluxes but after they peak the 3 × 3 drops off more rapidly with increasing pitch than does the static Hybrid CSCA. Dynamic 3 × 3 and dynamic Hybrid CSCAs both perform similarly well at all pixel pitches investigated here, producing higher BSE values than the 3 × 3 static case at all values tested, and outperforming static Hybrid CSCAs at all pitches in Figure 11 and all pitches below 400 in Figure 12.

**Figure 14:**
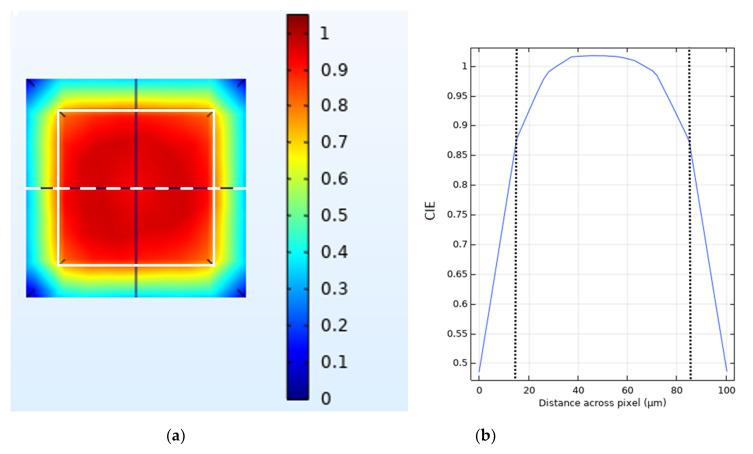
(**a**) Heat map representing charge induction efficiency (CIE) across a single pixel as generated and viewed in COMSOL. The plot represents a cut through taken perpendicular to the incident photon flux. The edges of the anode are marked by the white square superimposed on the image. (**b**) A plot of CIE vs. distance along a path taken across the centre of the pixel (the horizontal dashed white line in Figure 14a).

Figure 14 is included to facilitate discussions regarding the mechanisms responsible for trends identified in the above plots and will be referenced again in the discussion section. Figure 14a shows a cross section of the charge induction efficiency (CIE) for electrons taken at right angles to the direction of the incident photon beam. The CIE can be seen to be highly uniform in the space under the anode (demarcated by the central white square) but drops off rapidly as the distance from the anode increases. Figure 14b shows the CIE values taken along a line profile across the middle of Figure 14a. The CIE value can be seen to drop from ~87% at the edge of the anode (demarcated by the dotted lines) to <50% in the middle of the inter-anode spacing over a distance of only 15 µm.

## 4. Discussion

### 4.1. Absolute Detection Efficiency

The decreasing ADE score for detectors of increasing pitch when no CSCA is running can be explained by a simultaneous reduction in CSEs and increase in pulse pileup. CSEs reduce with increasing pixel pitch as larger pixels have a larger average path length that photons from Compton scattering or fluorescence processes need to travel to cross from one pixel to another. Pulse pileup increases because the cross section of the pixel with respect to the incident flux increases, thus resulting in a shorter average time between photon interactions. With this in mind, the effect of the various classes of CSCA on this metric can be considered and explained. Note that for the ADE metric only the energy bin that a count is assigned to is irrelevant. Hence, the 3 × 3 and Hybrid CSCAs can be treated as identical, as they both produce one count in response to an event within a 3 × 3 search area.

The first thing to note from Figure 4 is that all CSCAs result in lower ADE values than the No CSCA case. This is unsurprising given that the No CSCA case returns values significantly above that expected by the theoretical ADE when CSEs are absent, and CSCAs are designed to reduce CSEs. This implies that all CSCAs are effective at reducing CSEs to some extent, however the drop in ADE value will also likely result at least in part due to the CSCAs increasing pule pileup. Pulse pileup is increased primarily due to the NS value of the CSCAs, because an NxN CSCA represents an area to the incident flux N^2^ times greater than the No CSCA case.

To demonstrate the role played by pulse pileup it is helpful to consider cases where the flux is reduced (Figure 3) or increased (Figure 5) by an order of magnitude (with respect to Figure 4), and consequently pileup effects are reduced or increased respectively. From this it can be seen that pileup does contribute significantly to the drop in ADE value, as evidenced by the increasingly negative gradient of the ADE versus pitch plots for the various CSCAs, along with the progressively lower pitches at which the CSCAs begin to underperform compared with the theoretical ADE. Additionally, the ordering of the various data sets is consistent with NS being the primary determiner: 1 × 1 (No CSCA) produces the greatest ADE values, followed by 2 × 2 and then 3 × 3 CSCAs.

If pulse pileup alone was responsible for the apparent improvements in ADE then the NL of a CSCA should not make a difference, only the NS. This is not what is found however, with the dynamic 3 × 3 CSCA producing a markedly lower ADE value at low to moderate per pixel fluxes (Figure 3 and the lower pixel pitches of Figure 4). Similarly, dynamic 2 × 2 and dynamic 3 × 3 CSCAs producing higher ADE values at higher per pixel fluxes (the higher pitches in Figure 4 and all pitches Figure 5). Explanations for these two factors are offered, as follows.

First, the significantly lower ADE value of dynamic 3 × 3 CSCAs compared with static 3 × 3 CSCAs can be explained using a concept we refer to as “**geometric advantage**”. Geometric advantage refers to the higher probability of correcting for CSEs in dynamic 3 × 3 CSCAs caused by centering the search area around at least one of the events to be reconstructed. When charge is shared across touching pixels A and B in quick succession, the probability of both pixels being located within the same search area depends on the geometric nature of the pixel that the first of these events (A) is detected in. The 3 × 3 search areas contain three geometrically distinct pixel types: edges, corners, and centres. Whilst each pixel will have eight touching neighbours, only centre pixels will form search areas that contain all of these. Edge pixels will form search areas containing five neighbours, whilst corners will form search areas that only contain three. If pixel B does not lie in the same search area as A then the original event cannot be reconstructed, and two counts will result in the detector instead of one. Consequently, by always ensuring event A is in a centre pixel, dynamic 3 × 3 CSCAs maximise the chances of successfully reconstructing a CSE and so are better at reducing CSE than their static counterparts. Note that the same advantage is not afforded to 2 × 2 CSCAs as these only consist of corner type pixels, explaining why 2 × 2 CSCAs perform interchangeably at low fluxes where CSEs dominate.

**Figure 15:**
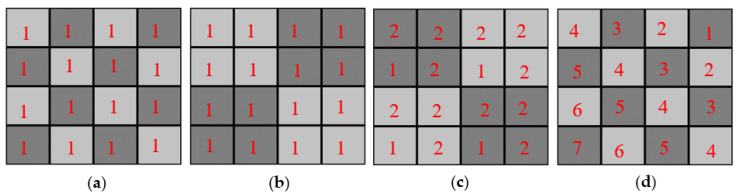
A 4 pixel × 4 pixel array can produce a range of different counts depending on its segmentation. The numbers in pixels refer to the order in which they are triggered. Pixels with the same number can be triggered in any order, so long as they are all triggered before higher numbered pixels. For the case where no CSCA is applied (**a**) the maximum count rate is equal to the number of pixels, 16. For a static 2 × 2 CSCA (**b**) the maximum count rate is equal to the number of neighbourhoods defined, 4. For a dynamic 2 × 2 CSCA however the maximum count rate depends on the order in which the pixels are triggered and can range from 4 (**c**) up to 16 (**d**). The more highly ordered state of the triggering required to produce 16 counts makes it less likely however, though the actually count rate will likely lie somewhere in between 4 and 16.

At higher fluxes (Figure 5), dynamic CSCAs show higher ADE values than their static counterparts for both the 2 × 2 and 3 × 3 NS types however, and we propose that this can be explained by something we call “**variable pixelation**”. This refers to the fact that, due to the way that search regions are defined, dynamic CSCAs can have higher maximum count rates than their static counterparts. Consider the case of a 4 × 4 pixel array shown in Figure 15. When no CSCA is running the maximum number of counts per cycle that this detector can report is 16 (Figure 15a), whilst for a static 2 × 2 CSCA the maximum number is 4 (Figure 15b). In both cases the maximum counts per cycle are equal to the fixed number of pixels available to report. Dynamic CSCA do not have a fixed number of pixels however, and with the right (extreme) stimulation can produce counts per cycle in the range of 4–16 (Figure 15c,d). In practice they are likely to produce some in-between value. To support this claim, a smaller series of Monte Carlo simulations was performed, considering a subset of the pixel pitches used in the main study. For these simulations, pixels were treated as having an infinitely long deadtime so that they could only record one count at most. Pixels were then selected at random (with a rectangular distribution) and neighbourhoods constructed according to the dynamic allocations rules used in the main study. The process was then repeated until all pixels had reported at least one count and the total number of counts, C_max_, was recorded. The process was repeated 10^4^ times and average values of C_max_ calculated. The results of this verification can be seen in Table 3.

It can be seen that dynamic 2 × 2 and 3 × 3 CSCAs are capable of maximum count rates 1.6 and 1.7 times greater than their static counterparts. This is an upper limit and strictly applies only to cases where the flux is so high that maximum count rates are reached. At low fluxes the chances of two neighbourhoods overlapping is considerably lower than at higher fluxes, and consequently the performance of the static and dynamic approaches should tend towards each other. This is the case for the 2 × 2 CSCAs, and at high fluxes (Figure 5), it also applies to the 3 × 3 CSCAs.

With these general trends in ADE established, the relative performance of the different CSCAs with respect to the metric of ADE can now be addressed. Figure 6 and Figure 7 shows the absolute deviation between each CSCA and the theoretical ADE as a function of pixel pitch. Lower values in these plots thus represent CSCAs that perform better with regards to the ADE metric. Considering first the 2 × 2 CSCAs, it can be seen that at low fluxes where pulse pileup is minimal (Figure 6 and lower pitches in Figure 7) the static and dynamic CSCAs perform similarly to each other, until they reach the maximum performance of the theoretical ADE, (at around 400 µm on Figure 7). This is because in this regime CSEs are the dominant factor in performance, and neither NL type for 2 × 2 has an advantage at correcting for these. At per pixel fluxes beyond this point (pitches above ~400 µm in Figure 7) the dominant detriment to ADE is pulse pileup, which affects dynamic CSCAs less significantly, causing them to deteriorate more slowly, and thus outperform their static counterparts. For the 3 × 3 CSCAs the geometric advantage afforded by dynamic NL approaches leads to significantly improved CSE reduction and consequently to lower ADE values. At high per pixel fluxes the dynamic CSCAs again have increased performance due to variable pixelation effects. In between these two regimes exists a region in which dynamic 3 × 3 CSCAs underperform their static counterparts (250–400 µm pitch in Figure 7). Indeed, the results shown in Figure 7 underscore the fact that there is no such thing as a “best” CSCA, or even that CSCAs are always better than the No CSCA case, but rather that the best approach will depend on the fluxes involved, pixel pitch and, as will be seen in the next section, the metric being assessed.

### 4.2. Absolute Photopeak Efficiency

In contrast to ADE, APE is a spectral metric, meaning that the energy assigned to a detected photon is relevant, and not just the total number of counts. As a result, the Hybrid and 3 × 3 CSCAs can be more clearly distinguished.

The general shape of the APE trends in Figure 9 is that of a peak, increasing with pitch up to a point and then decreasing with pitch beyond that point. The initial increase in APE with pixel pitch was previously shown to result from a reduction of CSEs [32], resulting in more incident photons being correctly assigned their full energy. The drop in APE at still higher pixel pitches is associated with pulse pileup effects, which lead to multiple photons being summed together, and thus the count being assigned to the higher energy bin 4 (coincidence bin) rather than the lower energy bin 3 (photopeak bin). The role of pileup in causing this decrease is evidenced by comparing Figure 8 with Figure 9, and noting that in a lower flux regime (and thus with less pileup) the curves peak at a higher pitch, with a higher APE value, and drop off much more gradually. Looking first at the effect of NS on CSCA performance we note that the rate of curvature for the different cases can be ranked as 3 × 3 > 2 × 2 > No CSCA. This is consistent with the interpretation that the drop off is caused by pileup as this is the same ordering as for effective pixel sizes, and effective pixel size should positively correlate with per pixel flux.

Considering the effect of NL instead reveals again that there is little difference between static and dynamic 2 × 2 CSCAs at low to medium fluxes, though again a slight advantage is observed for higher per pixel fluxes (>400 µm in Figure 9). This is again explained by the variable pixelation concept previous demonstrated; however, for a slightly different reason. As a consequence of overlapping search areas, the average effective pixel size of a dynamic CSCA is smaller than that of static CSCA. From Table 3 we can see that maximum count rates for 2 × 2 CSCAs is roughly 1.6 times larger than the static case, implying an average search area 1.6 times smaller, or roughly 2.5 pixels. At low fluxes search area overlap will be minimal, so the effective area will be approximately four pixels; however, as flux increases this will converge to the limit of 2.5 pixels. As smaller pixel sizes are less prone to pileup, these smaller pixels are better able to maintain their spectral performance, slowing the deterioration of the APE value and causing the static and dynamic 2 × 2 CSCA liens to diverge. A similar situation can be seen for the case of the 3 × 3 CSCAs, however notably for the Hybrid CSCAs the situation is reversed, with the static Hybrid CSCA outperforming the dynamic version at higher per pixel fluxes (from ~350 µm and greater in Figure 9). The reason proposed for this is that, unlike the 3 × 3 and 2 × 2 CSCAs, the Hybrid CSCA does not integrate charge from its whole search area but only from the 2 × 2 sub-search area that gives the highest resulting charge. The geometric advantage of a dynamic CSCA ensures that the centre pixel, which contributes to all possible sub-search areas, always contains some charge, and so maximises the resulting charge found. At lower fluxes, where pulse pileup is unlikely and CSEs are the dominant reasons that photon interactions fail to register in the photopeak bin, this combination of features is advantageous, correcting for CSEs better than the static Hybrid CSCA. At higher fluxes and larger pixel pitches however, where pileup is more likely than CSEs, this same feature means that the dynamic Hybrid CSCA will find coincident events, which are higher energy, more frequently than its static counterpart, and will actively select for them. That this is the case can be verified by looking at Figure 10, which shows RCC (a measure of pulse pileup) for the different CSCAs. As a result, the dynamic Hybrid CSCA actually produces a lower APE value than its static counterpart once pulse pileup becomes more probable than CSEs. This effect is referred to here as “**geometric disadvantage**”, and applies only to the Hybrid CSCA.

Considering next the relative heights of the peaks in Figure 8 and Figure 9, we can again see the importance of pulse pileup in determining which CSCA is most suitable for a given situation. At lower fluxes (Figure 8) all CSCAs result in a higher peak APE value than the No CSCA case and outperform the No CSCA case in at almost all pitches. In contrast, at higher fluxes (Figure 9) the No CSCA is the best option for over half over the pitches considered (>300 µm) and can achieve a peak APE value higher than any CSCA except for the dynamic 3 × 3/Hybrid. The fact that the dynamic 3 × 3/Hybrid CSCAs can produce the highest peak APE value under fluxes where the static 3 × 3/Hybrid CSCAs produce the lowest peak values shows that the geometric advantage is much larger than the pileup disadvantage at these per pixel fluxes. Being able to determine the ranges of fluxes and pitch over which this kind of thing happens is an advantage of simulated investigations such as this.

### 4.3. Relative Coincidence Counts

The results in Figure 10 should be unsurprising giving the discussion so far. The figure shows that the counts in bin 4, which must originate from pulse pileup, increase with increasing pixel pitch, which has been one of the cornerstones of the discussion so far. It is worth checking for consistency with the various other arguments made with regards the preceding metrics however, including the effect of variable pixelation, geometric advantage for dynamic 3 × 3 CSCAs and geometric disadvantage for the dynamic Hybrid CSCA.

The ordering of the CSCAs based on NS is consistent with expectations: the larger NS values lead to higher effective pixel sizes for intercepting incoming photons and so lead to increased pulse pileup (RCC counts). Considering just the static CSCAs for example, 3 × 3 > 2 × 2 > No CSCA, and the Hybrid CSCA, which searches a 3 × 3 area but only sums over a 2 × 2 area sits somewhere between the 3 × 3 and 2 × 2 cases, as would be expected.

Considering the effect of NL choice on RCC we again see that for the 2 × 2 and 3 × 3 (but not the Hybrid) CSCAs the dynamic CSCAs begin to diverge from their static counterparts at higher fluxes, experiencing lower RCC values. This is consistent with the variable pixelation idea proposed in this work as these systems can support higher count rates and have a slightly lower average pixel effective size, resulting in a lower susceptibility to pulse pileup. At low pixel pitches, where per pixel fluxes are lower and CSEs dominate, there is no difference between the static and dynamic cases for either the 2 × 2 or 3 × 3 CSCAs. This is because geometric advantage applies to correcting for CSEs by centring on the shared event, but does not significantly affect pulse pileup as the probability of this depends only on the effective number of pixels in the search area.

Finally, the static Hybrid CSCA can be seen to increase in RCC far more gradually than the dynamic Hybrid CSCA, consistent with the geometric advantage afforded to dynamic CSCAs becoming disadvantageous to Hybrid CSCAs as pileup becomes more significant. Again, this is an effect unique to the Hybrid CSCAs and can be explained by the dynamic approach ensuring that all sub-search areas contain at least the charge from the first detected event, increasing the chance that the highest sub-search area will contain a charge above threshold four.

### 4.4. Binned Spectral Efficiency

Figure 11 and Figure 12 show the relation between the spectral metric BSE and pixel pitch for the various CSCAs considered in this work at fluxes of 10^6^ and 10^7^ photons mm^−2^ s^−1^, respectively. As with APE, a peaked shape is present in the data, with reducing CSE leading to the rise from lower pixel pitches and pulse pileup responsible for the drop in value at higher pitches, and many of the general arguments made for APE apply for BSE too. BSE is more sensitive to the detrimental spectral effects of CSEs however, as the denominator in its definition is the sum of all counts in the detector rather than a constant like the total number of incident photons used for determining APE. As a result, a CSE that goes uncorrected not only fails to increment the numerator, but also increments the denominator multiple times. This increased sensitivity to CSE results in the peak BSE value occurring at a higher pitch than the peak APE value (250 vs. 200 µm for the dynamic 3 × 3 CSCA, and ~400 vs. ~250 for the 2 × 2 CSCAs). This is because reductions in CSE are more significant for BSE, resulting in more pulse pileup being required to offset the gains afforded by CSE reduction. It is notable that as a consequence of this increased sensitivity to CSE, the lower susceptibility to pulse pileup of the 2 × 2 CSCAs confers less of an advantage to them, and indeed over the flux ranges examined here the 2 × 2 CSCAs are never the best choice with regards to the BSE metric. This is significant as BSE is arguably the metric most often used in x-CSI applications.

For reasons already established when discussing the previous metrics, 3 × 3 and Hybrid CSCAs peak earlier than their 2 × 2 counterparts (due to larger effective pixel size), the dynamic 3 × 3 CSCA outperforms the static 3 × 3 CSCA (due to geometric advantage) and the dynamic Hybrid CSCA outperforms the static Hybrid CSCA at lower fluxes (Figure 11) and smaller pixel sizes (Figure 12 at pitches below ~ 350 µm) due to geometric advantage, but under performs it at higher per pixel fluxes (Figure 12 for pitch above ~350 µm) due to the geometric affects becoming disadvantageous.

One notable difference from other metrics is the lack of divergence between the 2 × 2 CSCAs of different NL type. It is proposed that this again results from the increased CSE sensitivity of this metric shifting the point at which pileup effects become significant in comparison to higher fluxes. In support of this, Figure 13 shows the BSE values for these CSCAs at 10^8^ photons mm^−2^ s^−1^, demonstrating that the dynamic 2 × 2 CSCA does indeed still experience an advantage over its static counterpart due to the previously discussed variable pixelation effects.

A major takeaway from the above discussion is that whilst it is often tempting to think of the benefits and drawbacks of CSCAs as resulting simply from their increase in effective pixel size, this is oversimplistic and a range of other factors can come into play such as geometric advantage and variable pixelation, and these factors can act in surprising ways when combined with more complex CSCAs such as the Hybrid model detailed here. Even without these effects (which all result from dynamic NL type CSCAs); however, it is still not the case that a static NxN CSCAs performs the same as pixels with a pitch N times greater and no CSCA. This can be seen from the data presented here by comparing BSE for example. Consider, for example, the 600 µm No CSCA, 300 µm static 2 × 2 and 200 µm static 3 × 3 cases at 10^7^ photons mm^−2^ s^−1^. Whilst these three arrangements represent an equivalent cross section to the incoming photon flux, they produce different BSE values: 62%, 58%, and 55% respectively. This is because BSE, like APE and RCC (which both show similar results), is a spectral metric and so is sensitive to the energy of assigned to a given count, not just its presence or absence. As the relation between incident photon energy and the energy assigned to an event is mediated by the CIE values at the points of interaction, it is worth considering an example CIE map used in this work and considering their implications to CSCAs. As can be seen from Figure 14, whilst the CIE for electrons is relatively high in the area directly under the anode, it drops off rather rapidly in the inter-anode spacing, meaning that photons liberating charge in these regions will register as at a lower energy than they actually are. As the inter-anode spacing is held constant in this work, 2 × 2 CSCAs and 3 × 3 CSCAs contain four and nine times as much of this inter-anode spacing, respectively, in their “effective pixel” areas. This explains why the spectral performance for the same effective pixel size is of the form No CSCA > static 2 × 2 > static 3 × 3. The spatial resolutions of these systems run in the opposite order, establishing that there is a trade-off between spectral performance and spatial resolution.

It should be noted that the current study is based on a range of assumptions which may limit the accuracy of any given quantitative predictions regarding a specific metric. In particular, whilst fluorescence photons and Compton scattering are modelled completely, charge cloud diffusion is modelled here only for its detrimental effect on the primary pixel of interaction. Consequently, the performance of some CSCAs may be underestimated at the smallest pixel sizes where charge clouds become comparable in size to the pixel pitch employed. Similarly, the CSCA process being modelled here is pre-thresholding, however electronic noise was not modelled in these simulations. The contribution of such noise would be expected to deteriorate the spectral resolution of the different CSCAs, with a higher impact on the 3 × 3 CSCAs than the 2 × 2 ort Hybrid CSCAs, due to the smaller number of pixels summed in these. Inclusion of this effect may therefore be expected to further differentiate the dynamic Hybrid and 3 × 3 CSCAs, with Hybrid CSCAs becoming more favourable.

Nevertheless, the current work demonstrates trends in CSCA performance as a function of pixel pitch and X-ray flux which will be of interest to the wider energy resolving/spectral photon counting community. Further, whilst quantitative conclusions should be treated with caution, the qualitative analysis performed in this work has revealed general mechanisms that will be informative for anyone aiming to design a CSCA.

## 5. Conclusions

The current work compared six different CSCAs using four different metrics: absolute detection efficiency (ADE), absolute photopeak efficiency (APE), relative coincidence counts (RCC), and binned spectral efficiency (BSE). Comparisons were considered at pixel pitches ranging from 100 µm to 600 µm in 50 µm steps, and at a range of fluxes spanning three orders of magnitude (10^6^–10^8^ photons mm^−2^ s^−1^). Contrary to common belief, effective pixel size was shown to be inadequate alone to explain the differences between the different CSCAs, with CSCAs utilizing the effective pixel size but different rules for locating them performing markedly differently. Considering first APE, novel concepts of geometric advantage and variable pixelation were proposed to describe these differences. Predictions based on these concepts, not previously proposed, were found to have consistent explanatory power when applied to trends found for BSE and RCC metrics. Geometric advantage applies to dynamic CSCAs greater than 2 × 2 in size. It refers to the increased probability of locating all pixels involved in a CSE within the same search area produced by centering the search area on the initial interaction site. Geometric advantage was consistently found for dynamic 3 × 3 CSCAs; however, the case was more complicated for dynamic Hybrid CSCAs, where the geometric factor could lead to an advantage or a disadvantage, depending on the metric assessed. Variable pixelation applies to all dynamic CSCAs and refers to the fact that effective pixel sizes begin to reduce in these CSCAs as the X-ray flux increases, as a result of defined search areas beginning to overlap. Together, geometric advantage, variable pixelation and effective pixel size were able to explain all of the observed trends.

The results of this work make clear that the answer to the question of which CSCA is best will depend upon the operating flux, pixel size and metric being considered, and that CSCAs do not always improve spectral performance compared with the same system running without CSCA, especially at higher pixel pitches and higher X-ray fluxes.

The work presented here represents a subset of a larger study into the effect of x-CSI system parameters on system performance. A previous publication [32] explored the effect of pixel pitch and thickness. Here we have examined how various classes of additive CSCAs compare at low to moderate medical fluxes. We plan to study next how additive and subtractive CSCAs compare at higher medically relevant fluxes.

## Figures and Tables

**Table 1:** CdTe material properties used for finite element models.

Parameter	Symbol	Value	Unit
Mobility, electrons	µ_e_	1100	cm^2^ V^−1^ s^−1^
Mobility, holes	µ_h_	100	cm^2^ V^−1^ s^−1^
Lifetime, electrons	τ_e_	3.0	µs
Lifetime, holes	τ_h_	2.0	µs
Density	Ρ	5850	kg m^−3^
Diffusion coefficient, electrons	D_e_	2.84 × 10^−3^	m^2^ s^−1^
Diffusion coefficient, holes	D_h_	2.58 × 10^−4^	m^2^ s^−1^
Relative permittivity	ε	11.0	-

**Table 2:** A list of the different CSCAs used in this work along with their NS and NL categories.

CSCA Label	NS	NL
2 × 2St+	2 × 2	Static
2 × 2Dy+	2 × 2	Dynamic
3 × 3St+	3 × 3	Static
3 × 3Dy+	3 × 3	Dynamic
HybridSt+	3 × 3 and 2 × 2	Static
HybridDy+	3 × 3 and 2 × 2	Dynamic

**Table 3:** The ratios of maximum count rates for dynamic and static of 2 × 2 and 3 × 3 CSCAs.

Array Size (pixels)	Equivalent Pixel Pitch (µm)	1 × 1 Max Count Rate	Static 2 × 2 Max Count Rate, S2C_max_	Dynamic 2 × 2 Max Count Rate, D2C_max_	D2C_max_/S2C_max_	Static 3 × 3 Max Count Rate	Dynamic 3 × 3 Max Count Rate	D2C_max_/S2C_max_
210 × 210	100	44,100	11,025	17,647	1.6	4900	8301	1.7
84 × 84	250	7056	1764	2840	1.6	784	1342	1.7
60 × 60	350	3600	900	1455	1.6	400	689	1.7
42 × 42	500	1764	441	716	1.6	196	341	1.7
